# Pricing policy for declining demand using item preservation technology

**DOI:** 10.1186/s40064-016-3627-x

**Published:** 2016-11-11

**Authors:** Uttam Kumar Khedlekar, Diwakar Shukla, Anubhav Namdeo

**Affiliations:** Department of Mathematics and Statistics, Dr Harisingh Gour Vishwavidyalaya (A Central University), Sagar, MP India

**Keywords:** Inventory, Preservation technology, Declining demand, Deterioration, Pricing, Replenishment cycle, 90B05, 90B30, 90B50

## Abstract

We have designed an inventory model for seasonal products in which deterioration can be controlled by item preservation technology investment. Demand for the product is considered price sensitive and decreases linearly. This study has shown that the profit is a concave function of optimal selling price, replenishment time and preservation cost parameter. We simultaneously determined the optimal selling price of the product, the replenishment cycle and the cost of item preservation technology. Additionally, this study has shown that there exists an optimal selling price and optimal preservation investment to maximize the profit for every business set-up. Finally, the model is illustrated by numerical examples and sensitive analysis of the optimal solution with respect to major parameters.

## Background

Retailers and manufacturers identify the importance of pricing policy in a competitive environment. Adequate pricing and marketing policies may boost the company’s bottom-line in such competition. Products such as fashion apparel, cosmetics, and winter wears become obsolete with the passing of time. Therefore, a pricing policy is required to ensure sale of the entire stock before entering the next cycle. Mostly, in a diminishing market, the manufacturers put their efforts into uplifting sales by reducing the price through media and pricing policy when demand declines. Fergany and Wakeel (2006) considered a probabilistic lost sales inventory system where order cost is a function of order quantity and lead time demand follows the normal distribution by using Lagrangian method. Chiu et al. (2016) developed two extended economic manufacturing quantity model to examine the production process, end product delivery and intra-supply chains.

Items that have a decreasing in quality or quantity over time are known as deteriorating items, and this process is called deterioration. One of the major assumptions of the inventory system is that the stock of goods can be indefinite, however some types of products either deteriorate or become obsolete with time, and the storage of such goods becomes difficult. This process can generally be seems in the case of commonly used goods such as fruits, vegetables, meat, perfumes, alcohols, gasoline, radioactive substances, photographic films and electronic components where deterioration is usually observed during their normal storage period. Banu and Mondal (2016) considered an economic order quantity model for deteriorating items where the demand function is linked with the customers’ credit period and the duration of policy of such credit period is exponential in nature.

Blackburn and Scudder (2009) examined warehouse temperature according to warehouse capacity constraints. Fergany (2016) proposed a probabilistic multi-item, single-source inventory model with varying mixture shortage costs under two restrictions. Kouki et al. (2013) reveal that a continuous temperature control policy can be very efficient for inventory management. Widyadana and Wee (2010) devised an EPQ model for deteriorating items by considering stochastic demand. In this model, lost sales will occur when the machine unavailability time is longer than the non-production time. Khedlekar et al. (2014) developed a production inventory model for deteriorating items with production disruption and analyzed the system under different situations. Chandel and Khedlekar (2013) designed an integrated inventory model to optimize the total expenditure for warehouse set-up. Shukla et al. (2012) considered an optimal selling price for optimal profit in a certain business set-up and concluded that if demand for products is less price-sensitive, optimal profit will be greater but permit less price-setting. Khedlekar and Shukla (2012) developed a dynamic pricing model for products with logarithmic decline price-sensitive demand and found that *β* is the most significant parameter affecting optimal profit and the respective number of price settings.

Chen and Zhang (2010) developed a three-echelon supply chain system consisting of suppliers, manufacturers and customers under demand disruptions by using a jump-diffusion model. Furthermore, an improved analytical hierarchy process (AHP) studied for selection of the best suppliers based on quantitative factors such as optimal long-term total cost. The objective was to minimize the total cost under different demand disruption scenarios. Banerjee and Roy (2010) formulated a multi-objective inventory model for both exponential and uniform lead time where demand was taken, and it was found that fuzzy optimization obtained better results.

Roy and Chaudhuri (2011) introduced an economic production lot-sized model where the production rate depends on the stock and selling price per unit. In this model, deterioration is assumed as a constant fraction allowing no shortages. Balkhi and Bakry (2009) devised a dynamic inventory model of deteriorating items in which each of the production, demand and deterioration rates were assumed to be general functions of time. Both inflation and time value of money were incorporated, and the optimal stopping and restarting production times in any cycle could be determined.

Giri et al. (2003) extended the economic lot-scheduling problem where the production follows a normal distribution. Sarkar and Moon (2011) designed a classical EPQ model with stochastic demand under the effect of inflation. The model is described by considering a general distribution function. Chang et al. (2010) determined an EOQ model for deteriorating items by assuming that demand rate depends not only on the on-display stock level but also on the selling price per unit as well as the shelf or display space. They formulated two types of mathematical models to manifest the extended EOQ models for maximizing profits and derived the algorithms to find the optimal solution. The recent development of some EOQ models [Shukla and Khedlekar (2015), He and He (2010) and Zang et al. (2014)] has been important in the current competitive market. Chang and Dye (1999) modeled a business with a constant deterioration rate and time-varying demand with the assumption that the shortages were partially backlogged and the backlogging rate was a decreasing function of the waiting time for the next replenishment. A continuous production control inventory model for deteriorating items with shortages was developed by Samanta and Roy (2004), they assumed demand and production rates are constant and the deterioration of an item follows an exponential distribution. Hence, this model is applicable to food items, drugs, pharmaceuticals, etc. Dye and Hsieh (2012) extended Hsu et al.’s (2010) model by incorporating a time-varying deterioration rate. He and Huang (2013) devised a model for deteriorating seasonal products whose deterioration rate can be controlled by investing in preservation efforts. This model suggests preservation technology investment and pricing strategies for deteriorating seasonal products. Zang et al. (2014) designed an inventory model wherein demand is dependent on selling price and time and deterioration can be controlled by preservation technology. There are numerous studies on inventory models for deteriorating items under different conditions, such as Goswami and Chaudhuri (1991), Alamri and Balkhi (2007), Khedlekar et al. (2012), Chung and Huang (2007), Ouyang et al. (2005), and Kumar and Sharma (2012).

The study of a deteriorating inventory model is of extreme importance for the smooth and efficient running of any business organization. Deterioration may occur when goods decay, damage, spoil, or evaporate, or are obsolete or pilfered, all of which lead to loss of utility or loss of marginal value of commodities. The deterioration rate is assumed to be constant at the beginning of inventory modeling, but it varies according to time, weather, season, introduction of new technologies or new generation, such as grease, oil, petrol, alcohol, pharmaceutical substances, medicine, iron products, vegetables, perfumes, computer apparels, mobiles, and cosmetics. We apply preservation technology to protect the product from such types of deterioration, but this effort adds an additional cost to the total cost, known as the preservation technology investment cost.

The assumption of this study is that if we invest an optimal preservation cost *u*, the reduced deterioration rate becomes $\theta \left( {1 - f\left( u \right)} \right)$, which maximizes the total profit TP. Similarly, the demand rate is generally taken to be constant, but demand is never constant in reality. Since demand is affected by time, season and weather, it is a very sensitive factor related to any business management. Market price is highly related to market demand, so another assumption is that demand is considered time-dependent and linearly related to market price. Thus, here we assume the demand $D\left( {p,t} \right) = \alpha - at - \beta p$ is price sensitive, time dependent and linearly declining. Since the time horizon is taken as infinite in this study, we determined the optimal time for replenishment.

The main goal of this study is to determine the optimal selling price, the optimal length of the replenishment cycle and the optimal preservation technology investment simultaneously, such that the total profit per unit time is maximized.

The rest of the paper is organized as follows: In section “Assumption and notation”, the assumption and notations are presented. In section “Mathematical model”, a mathematical model to maximize the total profit per unit time is established, and three propositions are developed. In section “Numerical example”, two numerical examples are given. In section “Discussions”, the conclusion is presented to summarize the outputs.

## Assumption and notation

We assume that market demand is linearly related to market price and that it cannot be backlogged. Deteriorated products have no value, and the lead time is considered to be zero. The proportion of reduced deterioration rate *f*(*u*) is considered to be a continuous, increasing and concave function of item preservation technology parameter *u*, there for, *f*′(*u*) > 0, *f* ″(*u*) < 0 and *f*(0) = 0. Price sensitive, non-negative and exponentially declined demand rate $D\left( {p,t} \right) = \alpha - at - \beta p$ is considered in this model, where *α* > 0 the initial is demand and *β* > 0 is a price sensitive parameter. The model in this paper is built on the following assumptions:

*p*: Market price per unit, where *p* > *c*
*h*: Inventory holding cost unit per unit time
*T*: Length of the replenishment cycle, where the end of the cycle inventory is zero
*I(t)*: Inventory level at time *t*
*Q*: Order quantity per cycle
*K*: Replenishment cost per order
*c*: Purchasing cost per unit
*D*(*p,t*): Demand rate is a function of both price and time
*θ*: Deterioration rate, where 0 ≤ *θ* ≤ 1
*u*: Preservation technology investment parameter per unit time to reduce the deterioration rate
*f*(*u*): Proportion of reduced deterioration rate, where 0 ≤ *f*(*u*) ≤ 1
*TP*(*T, p, u*): Total profit per unit time

## Mathematical model

**Fig. 1:**
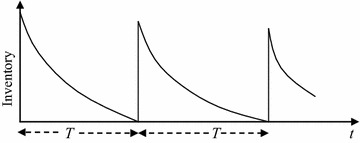
Graphical representation of the inventory system

We have considered a single retailer inventory model of seasonal products. Deterioration is reduced by preservation technology investment. The decision variable is the selling price of the product and the preservation technology investment parameter *u*. If $\theta$ is the deterioration rate and *f*(*u*) is the proportion of reduced deterioration rate by investing preservation technology costs, then the inventory at time *t* is shown in Fig. 1 and follows this differential equation:

1 $$\frac{\partial I\left( t \right)}{\partial t} + \theta \left( {1 - f\left( u \right)} \right)I\left( t \right) = - D\left( {p,t} \right)\quad {\text{where}}\; D\left( {p,t} \right) = \alpha - at - \beta p,\;0 \le t \le T$$

The boundary condition *I(T)* = *0*, leads

2 $$I\left( t \right) = \frac{{\left( {\alpha - at - \beta p} \right)\,e^{{\theta \left( {1 - f\left( u \right)} \right)\left( {T - t} \right)}} }}{{\theta \left( {1 - f\left( u \right)} \right)}} - \frac{\alpha - at - \beta p}{{\theta \left( {1 - f\left( u \right)} \right)}} - \frac{a}{{\theta^{2} \left( {1 - f\left( u \right)} \right)^{2} }} + \frac{{ae^{{\theta \left( {1 - f\left( u \right)} \right)\left( {T - t} \right)}} }}{{\theta^{2} \left( {1 - f\left( u \right)} \right)^{2} }}$$

The on-hand inventory *Q* will be

3 $$Q = \frac{{\left( {\alpha - aT - \beta p} \right)e^{{\theta \left( {1 - f\left( u \right)} \right)T}} }}{{\theta \left( {1 - f\left( u \right)} \right)}} - \frac{\alpha - \beta p}{{\theta \left( {1 - f\left( u \right)} \right)}} - \frac{a}{{\theta^{2} \left( {1 - f\left( u \right)} \right)^{2} }} + \frac{{ae^{{\theta \left( {1 - f\left( u \right)} \right)T}} }}{{\theta^{2} \left( {1 - f\left( u \right)} \right)^{2} }}$$

The total profit *TP*(*T, p, u*) of the season can be formulated as

4 $$\begin{aligned} & TP\left( {T, \, p, \, u} \right) = {\text{ Sales revenue }}\left( R \right) \, {-}{\text{ Purchasing cost }}\left( {c_{p} } \right) \, {-}{\text{ Inventory holding cost }}\left( {c_{h} } \right) \\ & \quad {\text{Preservation cost }}\left( {I_{o} } \right) \, - {\text{ Replenishment cost }}(K) \\ \end{aligned}$$

### Sales revenue

The total revenue in time *T* can be formulated as

$$R = p\left( {\alpha T - \frac{{aT^{2} }}{2} - \beta pT} \right)$$

### Purchasing cost

According to Eq. (3), we know the order quantity. Thus, the total purchasing cost can be formulated as

$$c_{p} = cQ$$

### Inventory holding cost

The formulation of the total inventory holding cost is

$$c_{h} = - \frac{{h\left( {\alpha T - \frac{{aT^{2} }}{2} - \beta pT} \right)}}{x} - \frac{{h\left( {\alpha - \beta p} \right)}}{{x^{2} }}\left( {1 - e^{xT} } \right)$$

### Preservation cost

Preservation technology investment depends on the cycle length. For the inventory cycle *T*, the preservation technology investment cost is

$$I_{0} = uT$$

### Replenishment cost

The replenishment cost is

$$c_{0} = K$$

by Eq. (4), the total profit function per unit time is

5 $$\begin{aligned} & TP\left( {T,\,p,\,u} \right) = \frac{p}{T}\left( {\alpha T - \frac{{aT^{2} }}{2} - \beta pT} \right) - \frac{c}{T}\left[ { - \frac{\alpha - \beta p}{x} - \frac{a}{{x^{2} }} + \frac{{\left( {\alpha - aT - \beta p} \right)e^{xT} }}{x} + \frac{{ae^{xT} }}{{x^{2} }}} \right] \\ & \quad + \frac{h}{Tx}\left( {\alpha T - \frac{{aT^{2} }}{2} - \beta pT} \right) + \frac{h}{{Tx^{2} }}\left( {\alpha - \beta p} \right)\left( {1 - e^{xT} } \right) - u - \frac{K}{T},\quad {\text{where}}\,x = \theta \left( {1 - f\left( u \right)} \right) \\ \end{aligned}$$

#### **Proposition 1**

There exists a unique p* that maximizes the profit function $TP\left( {T,\,p,\,u} \right)$ *for fixed T and optimal u.*

#### *Proof*

The first and second partial derivatives of the profit function $TP\left( {T,\,p,\,u} \right)$ with respect to *p* are as follows:

$$\frac{{dTP\left( {T,\,p,\,u} \right)}}{dp} = \alpha - \frac{aT}{2} - 2\beta p - \frac{c}{T}\left( {\frac{\beta }{x} - \frac{{\beta e^{xT} }}{x}} \right) - \frac{h\beta }{x} - \frac{h\beta }{{Tx^{2} }}\left( {1 - e^{xT} } \right)$$

Let $\frac{{dTP\left( {T,\,p,\,u} \right)}}{dp}$ be zero and solve for optimal p*, we have

6 $$p* = \frac{\alpha }{2\beta } - \frac{aT}{4\beta } - \frac{1}{2xT}\left( {c + \frac{h}{x}} \right)\left( {1 - e^{xT} } \right) - \frac{h}{2x}$$

At point *p* = p*, we have

$$\frac{{\partial^{2} TP\left( {T,\,p,\,u} \right)}}{{\partial p^{2} }} = - 2\beta < 0$$

Thus, p* is the optimal market price that maximizes the profit function for fixed *T* and optimal *u*.

#### **Proposition 2**

*The profit function* $TP(T,\,p,\,u)$ *is concave in the replenishment cycle T.*

#### *Proof*

The first and second partial derivatives of the profit function $TP(T,\,p,\,u)$ with respect to *T* are as follows, where ${\text{x }} = \, \theta \left( {1 - f(u)} \right)$

and

$$\begin{aligned} \frac{{\partial^{2} TP(T,\,p,\,u)}}{{\partial T^{2} }} & = \frac{ac}{{T^{3} }}\left( {\frac{\alpha - \beta p}{x} + \frac{a}{{x^{2} }}} \right) \\ & \quad - \frac{c}{x}\left[ {\left( {\alpha - \beta p - aT} \right)\frac{{x^{2} }}{T} - 2\left( {\alpha - \beta p} \right)\frac{x}{{T^{2} }} + \frac{2}{{T^{3} }}\left( {\alpha - \beta p} \right)} \right]e^{xT} \\ & \quad - \frac{ac}{{x^{2} }}\left( {\frac{{x^{2} }}{T} - \frac{2x}{{T^{2} }} + \frac{2}{{T^{3} }}} \right)e^{xT} \\ & \quad - \frac{h}{{x^{2} }}\left( {\alpha - \beta p} \right)\left[ {\frac{{x^{2} }}{T} - \frac{2x}{{T^{2} }} + \frac{2}{{T^{3} }}\left( {1 - e^{ - xT} } \right)} \right]e^{xT} < 0 \\ \end{aligned}$$

Hence, the total profit function is concave in *T*. Thus, there exists a unique optimal replenishment time *T* ^*^ that maximizes $TP(T,\,p,\,u)$, and the optimal *T* ^*^ can be obtained by solving $\frac{\partial TP(T,\,p,\,u)}{\partial T} = 0$.

#### **Proposition 3**

*For any given feasible p and T, there exists a unique optimal preservation technology investment u* ^*^ *that maximizes TP*(*p,T,u*).

#### *Proof*

The first and second partial derivatives of the profit function *TP*(*p,T,u*) with respect to *u* are

$$\begin{aligned} & \frac{\partial TP(p,T,u)}{\partial u} = \left( {\frac{{c\left( {\alpha - \beta p} \right)}}{T\theta } + \frac{{h\left( {\alpha - \frac{aT}{2} - \beta p} \right)}}{\theta }} \right)\frac{f'(u)}{{\left( {1 - f(u)} \right)^{2} }} + \frac{2ac}{{T\theta^{2} }}\frac{f'(u)}{{\left( {1 - f(u)} \right)^{3} }} \\ & \quad c\left( {\alpha - aT - \beta p} \right)\frac{{f'(u)e^{{\theta \left( {1 - f(u)} \right)T}} }}{{\left( {1 - f(u)} \right)}} - \left( {\frac{c}{T\theta }\left( {\alpha - aT - \beta } \right) - \frac{{h\left( {\alpha - \beta p} \right)}}{\theta } - \frac{ac}{\theta }} \right)\frac{{f'(u)e^{{\theta \left( {1 - f(u)} \right)T}} }}{{\left( {1 - f(u)} \right)^{2} }} \\ & \quad - \frac{2ac}{{T\theta^{2} }}\frac{{f'(u)e^{{\theta \left( {1 - f(u)} \right)T}} }}{{\left( {1 - f(u)} \right)^{3} }} - \frac{{2h\left( {\alpha - \beta p} \right)}}{\theta }\frac{f'(u)}{{\left( {1 - f(u)} \right)^{2} }} - 1 \\ & \quad - \frac{{f'^{2} (u)}}{{\left( {1 - f(u)} \right)^{3} }}\frac{{\left( {\alpha - \beta p} \right)\left( {c - hT} \right)}}{T\theta }e^{{\theta \left( {1 - f(u)} \right)T}} \\ \end{aligned}$$

Since *f*′*(u)* > 0 and *f* ″*(u)* < 0, it is clear from the above equation that $\frac{{\partial^{2} TP(p,T,u)}}{{\partial u{}^{2}}} < 0$.

Hence, the total profit is a concave function of the preservation cost.

## Numerical example

### Example 1

In this example, we have considered that the reduced deterioration rateis $$f(u) = 1 - e^{{ - \gamma {\text{ u}}}}$$, $$\gamma > 0$$. The parametric values of the inventory system are as follows: *K* = $10 per order, *c* = $10 per unit, *h* = $0.5 per unit per month, *u* = $5 per unit time, *θ* = 0.01, *α* = 500, *β* = 0.5, *γ* = 0.05, and *ε* = 0.0001. Then, the optimal selling price per unit is p* = $12.6834, the optimal replenishment time *T* ^*^ = 0.712, the total profit per unit time $$TP(T^{ * } ,\,p^{ * } ,\,u^{ * } )$$ = $416 and the order quantity *Q* ^*^ = 26.

Next, we study the effects of changing the values of the system parameters on the p*, T*, $$TP(T^{ * } ,\,p^{ * } ,\,u^{ * } )$$ and *Q* ^*^. A sensitive analysis is performed by changing one parameter value by +40%, +20%, −20%, and −40%, and keeping the remaining parameters unchanged. For the simulation, the initial data are taken as in example 1, except the parameters *c* = $5, *h* = $1, *u* = $10, *α* = 100, and *β* = 5. The computational results are illustrated in Table 1.

**Table 1:** Sensitivity analysis with respect to the major parameters

Input parameters	Output parameters	−40%	−20%	0%	20%	40%
*α*	*p*	8.77	10.72	12.69	14.67	16.65
	*Q*	17	22	26	30	33
	*T*	1.050	0.830	0.712	0.633	0.576
	*TP*	103	239	416	633	891
*β*	*p*	19.34	15.18	12.69	11.03	9.85
	*Q*	28	27	26	25	24
	*T*	0.67	0.69	0.712	0.74	0.77
	*TP*	765	549	416	325	258
*γ*	*p*	12.685	12.684	12.6833	12.683	12.682
	*Q*	26	26	26	26	26
	*T*	0.712	0.712	0.712	0.711	0.711
	*TP*	416.06	416.03	416	415.97	415.90
*h*	*p*	12.65	12.67	12.68	12.70	12.72
	*Q*	34	29	26	24	22
	*T*	0.910	0.792	0.712	0.654	0.606
	*TP*	420.24	418.18	416	413.79	411.70

## Discussions

Based on the results in Table 1, we observe the following facts:

The increasing initial demand *α*, the optimal total profit per unit time $TP(T^{ * } ,\,p^{ * } ,\,u^{ * } )$, the optimal selling price p*, and optimal order quantity *Q* ^*^ increase while the optimal replenishment time *T* ^*^decreases. As a result, we have to maintain a high initial demand by ordering more quantity per replenishment cycle and shortening the replenishment cycle. Moreover, if the scaling factor *α* is getting low, then the enterprise terminates the order.

When the price sensitivity parameter *β* increases, the optimal $TP(T^{ * } ,\,p^{ * } ,\,u^{ * } )$, p* and *Q* ^*^ decreases while the optimal length of replenishment cycle *T* ^*^ increases. Thus, demand declines with a higher market price. To maintain this demand, we must reduce the optimal selling price.

When increasing value of parameter *γ*, the total profit per unit time $TP(T^{ * } ,\,p^{ * } ,\,u^{ * } )$ and the optimal selling price p*decrease marginally. Therefore, this parameter does not change the model output.

When the holding cost per unit time *h* increases, the optimal selling price p* increases, while the optimal order quantity *Q* ^*^, the optimal replenishment cycle *T* ^*^and the total profit $TP(T^{ * } ,\,p^{ * } ,\,u^{ * } )$ decrease. The minimum holding cost leads to maximizing the total profit.

### Example 2

In this example, the parameters are the same as those in example 1, except for the deterioration rate *θ*. For the given value of *θ*(=0.01, 0.02, 0.03, 0.04, 0.05, 0.06, 0.07, 0.08, 0.09, 0.1), we find the corresponding optimal value *p* ^*^, *Q* ^*^ *, T* ^*^and $TP(T^{ * } ,\,p^{ * } ,\,u^{ * } )$. The computational results are shown in Table 2.

**Table 2:** Sensitivity analysis with respect to *θ*

*θ*	T	P	*TP*	Q
0.01	0.712	12.6833	416.00	26.10
0.02	0.708	12.6879	416.20	26.00
0.03	0.701	12.6914	416.30	25.75
0.04	0.691	12.6945	416.33	25.50
0.05	0.682	12.6974	416.36	25.16
0.06	0.773	12.7002	415.38	24.87
0.07	0.664	12.7028	414.40	24.57
0.08	0.656	12.705	413.39	24.31
0.09	0.648	12.708	412.38	24.05
0.10	0.639	12.711	411.38	23.75

## Discussions

**Fig. 2:**
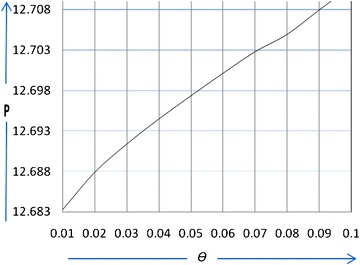
The effect of *θ* on the selling price (*p*)

**Fig. 3:**
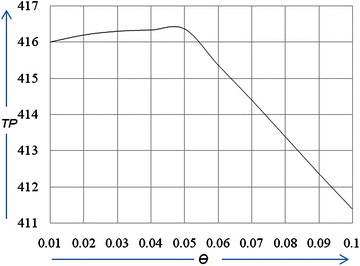
The effect of *θ* on total profit (*TP*)

When the deterioration rate increases and reaches a certain value, it is obvious that we will increase the preservation technology investment per unit time according to the deterioration rate. We observed from Table 2, that as the deterioration rate increases, the optimal replenishment cycle *T* ^*^ decreases; therefore, we must order in as small of lots as possible. Additionally, the optimal order quantity *Q* ^*^ will decrease because the goods will either deteriorate or sell out due to the successive deterioration rate with a lower selling price. After a certain point, as the deterioration rate increases, the total profit per unit time $TP(T^{ * } ,\,p^{ * } ,\,u^{ * } )$ will decrease; this means that if the deterioration rate is relatively large, the enterprise will invest more funds in preservation technology to reduce it. As shown in Figs. 2 and 3, to maintain profits due to increasing deterioration, we must increase the selling price *p*.

## Conclusion

The concept of item preservation technology is developed for price-sensitive demand. This paper concludes that there is a unique optimal selling price, optimal length of replenishment cycle and optimal item preservation technology investment for obtaining optimal profit. Numerical examples are provided to illustrate the proposed model. Sensitivity analysis is provided with respect to some key parameters to manage the system. Ordering of small lots is advised for retailers to reduce deterioration. The incorporation of preservation technology investment may significantly reduce the deterioration. Sensitivity analysis reveals that if initial demand increases or decreases, then we must adapt by ordering either more quantities per cycle or by reducing orders accordingly. Higher market price affects the market demands. We need to keep market demand progressive by reducing the selling price accordingly. Moreover, to maximize the model output, we need to keep the numerical value for parameter *γ* as low as possible. There is a need for balance between holding costs and preservation technology investment costs in order to obtain maximum profit.

One can extend this model for stochastic demand and variable holding costs. The proposed model can also be designed in a fuzzy environment. The theory can also be applied to growing and deckling markets separately with variable deterioration rates.
